# Digital Breast Tomosynthesis Changes Management in Patients Seen at a Tertiary Care Breast Center

**DOI:** 10.1155/2014/658929

**Published:** 2014-03-03

**Authors:** L. Margolies, A. Cohen, E. Sonnenblick, J. Mandeli, P. H. Schmidt, J. Szabo, N. Patel, G. Hermann, C. Weltz, E. Port

**Affiliations:** ^1^Department of Radiology, Icahn School of Medicine at Mount Sinai, One Gustave Levy Place, P.O. Box 1234, New York, NY 10029, USA; ^2^Department of Surgery, Icahn School of Medicine at Mount Sinai, One Gustave Levy Place, P.O. Box 1148, New York, NY 10029, USA; ^3^Department of Preventive Medicine, Icahn School of Medicine at Mount Sinai, 17 East 102 Street, New York, NY 10029, USA

## Abstract

*Objectives.* To study factors that predict changes in management with digital breast tomosynthesis (DBT). *Methods.* The Institutional Review Board approved this HIPAA compliant study. 996 patients had DBT with full field digital mammography (FFDM). Univariate analysis evaluated predictors of management change and cancer detection. *Results.* DBT changed management in 109 of 996 (11%); 77 (71%) required less imaging. Recalled patients after abnormal FFDM screen were most likely to have management change—25% (24 of 97 patients) compared to 8% (13/163) of symptomatic patients and 10% (72/736) of screening patients (*P* < 0.001). Dense breasted patients had a higher likelihood of having DBT change management: 13% (68/526) compared to 9% (41/470) (*P* = 0.03). Of the 996 patients, 19 (2%) were diagnosed with breast cancer. 15 cancers (83%) were seen on FFDM and DBT; 3 (17%) were diagnosed after DBT (0.3%, 95%CI: 0.1–0.9%). One recurrence was in the skin and was not seen on DBT nor was it seen on FFDM. The increase in cancer detection rate was 17% for asymptomatic patients, 0% for symptomatic patients, and 100% for recalled patients. *Conclusions.* DBT increased cancer detection rate by 20% and decreased the recall rate in 8–25%. *Advances in Knowledge.* DBT led to a doubling of the cancer detection rate in recalled patients.

## 1. Introduction

Prospective trials of screening mammography demonstrate a reduction in breast cancer mortality [1, 2] yet concerns regarding false positive and false negative findings generate controversy [3]. In parallel with efforts to define populations who may benefit from supplemental ultrasound or MRI screening [4–8] technical innovations to improve mammography's sensitivity and specificity have included full field digital mammography (FFDM) [9, 10], computer aided detection (CAD) [11], and digital breast tomosynthesis (DBT) [12].

DBT images potentially reduce false positives caused by overlapping tissues which can mimic cancer while simultaneously reducing false negatives caused by tissue overlap which could obscure malignancy. Thus, DBT improves the sensitivity of screening mammography compared with FFDM while decreasing recall rates [13–15]. Much work investigating DBT, however, has been retrospective or performed on enriched data sets [16–18] and there are few studies on the role of DBT in populations at elevated risk for breast cancer. In a meta-analysis of 14 studies, no data was available regarding the clinical variables associated with enhanced cancer detection by DBT [13]. Here, we investigated groups of patients for whom DBT altered management. We sought to prospectively determine the effect on patient management of adding DBT to FFDM in asymptomatic, symptomatic, and recalled patients presenting to a dedicated breast imaging center and sought to identify clinical factors which could predict the added value of DBT.

## 2. Methods and Materials

This study was compliant with the Health Insurance Portability and Accountability Act (HIPAA) and was approved by the Institutional Review Board. A waiver of informed consent was granted.

From January 6, 2012, to November 28, 2012, all patients who presented or were referred to our comprehensive breast center for screening or diagnostic mammography were offered or randomly selected for the combined FFDM/DBT exam at no extra cost. During the prospective study period 1011 DBT examinations were performed; 15 patients were excluded due to recently diagnosed breast cancer, leaving 996 patients in the cohort.

The imaging was obtained and interpreted using standard clinical protocol including the use of CAD for FFDM (R2 Image Checker Version 9.3 Hologic Corporation, Bedford, MA). All images were obtained on a commercially available DBT Hologic Selenia Dimensions system (Hologic Corporation, Bedford, MA). All breasts were imaging in the craniocaudal (CC) and medial lateral oblique position (MLO). The FFDM and DBT images were obtained in the same compression using automatic exposure control; the mA, kVp, and dose were automatically selected by the mammography equipment. The DBT acquisition uses 15 projection images that were reconstructed into 1 mm thin sections for viewing on a Hologic workstation. Both FFDM and DBT images were viewed on the same Hologic workstations. One of four board certified MQSA certified radiologists with 3 to 10 years of FFDM experience and 8–20 months of DBT experience interpreted the images. Prior images and clinical history were made available.

A demographic, clinical data and result sheet were provided to the reading radiologist for each patient undergoing DBT. The radiologist prospectively recorded whether the patient presented for asymptomatic screening or symptomatic diagnostic evaluation or was recalled from an abnormal FFDM screening exam. Other clinical and demographic data including personal or family history of breast cancer, history of prior biopsy, and pathology results (if available) were recorded.

The radiologist first interpreted the FFDM images blinded to the DBT images and assigned one of the standard Breast Imaging Reporting and Database System (BI-RADS) [19] codes for breast density: fatty, scattered fibroglandular elements, heterogeneously dense or extremely dense. The same radiologist then assigned a BI-RADS assessment code and made recommendations for further evaluation if needed. The radiologist then accessed the DBT images, interpreted the DBT study, and recorded change in recommendations if any. Results were entered into a HIPAA compliant database.

Change in management was defined as a change in recommendation for additional imaging, biopsy, or follow-up interval. Cancer detection rates before (FFDM alone) and after the addition of DBT were assessed.

Univariate analysis was performed using a nonparametric chi-square (Pearson's chi-square) at the *α* = 0.05 level. Risk factors were tested as independent predictors of change in clinical management due to the addition of DBT. Proportions were compared using the *z* ratio for difference between two independent proportions. Change in management was assigned per patient with no distinction made between unilateral or bilateral change. Cancer detection rates were calculated for FFDM and DBT. Change in cancer detection rate due to the addition of DBT was calculated. Statistical analysis was limited to a 95% Confidence Interval due to lack of independence between subjects in the FFDM and DBT groups.

## 3. Results

The patient population presenting to our comprehensive breast cancer center was heterogeneous in terms of factors relevant to breast cancer risk but overall represented a cohort at increased risk. Of the 996 cases, 207 (21%) had a history of breast cancer and 68 (6.8%) had a mastectomy and underwent unilateral imaging. (see Tables 1(a) and 1(b)). A total of 711 (71.4%) screening exams and 285 (28.6%) diagnostic exams were performed in 996 patients. Screening exams were performed in 330 (46.4%) women at normal risk, 188 (26.4%) patients with a personal history of breast cancer, 185 (26.1%) patients at increased risk for breast cancer due to a family history of breast cancer, a history of atypical lesions, and/or BRCA mutation, and 8 (1.1%) patients with a distant history of breast intervention. A total of 285 (28.6%) diagnostic exams were performed on 168 (58.7%) patients presenting with symptoms, 97 (34.0%) recalled for additional imaging, and 21 (7.3%) presenting for short term followup (see Table 1(b)).

### 3.1. Impact of DBT on Management

For 887 of 996 (89%) patients, results and recommendations did not change after DBT imaging, whereas DBT resulted in a change in management in 109 of 996 (11%) patients (See Table 2). 79 of the 109 whose management was changed had bilateral imaging, and 30 had unilateral exams, due to previous contralateral mastectomy, short term followup for benign biopsy, and/or abnormal imaging, or because recall was only indicated for one side. Change in management affected 1 breast in 105 patients and both breasts in 4 patients. Of the 4 patients with bilateral changes, 2 patients had additional studies requested for one side, and fewer studies requested on the other side. The other 2 patients did not need recall after DBT was reviewed.

Of the 109 patients who had changes in management, 77 (71%) did not need to be recalled for a diagnostic evaluation after DBT. Thus 77 of 996 (7%) of all patients had a change in management requiring less additional imaging. For these 77 patients, 91 exams that would have been performed based on the FFDM were cancelled after the DBT exam was reviewed. The 91 cancelled exams included additional mammographic images in 53 patients, ultrasound in 10 patients, and both additional views and ultrasound in 14 patients (28 exams). In this group DBT served as the additional imaging which was included in the screening exam and, thus, the patient was not recalled for diagnostic imaging.

The 77 patients who would have been recalled had it not been for DBT were followed for an average of 17 months (range: 13–22 months). Upon review of their electronic medical record, no cancers have occurred in these 77 women. 48 did have normal imaging subsequent to the index exam. 29 have not returned for a followup imaging exam to date. One patient presented to dermatology 2 months after the mammogram with a 3 mm cutaneous metastatic deposit. This was not seen in retrospect on FFDM or DBT nor was cancer found in breast tissue. No other patients have been diagnosed with breast cancer; that is, to date no false negatives have resulted from the changes in management based on DBT.

Thirty-four of the 109 (31%) patients had a change in management where additional imaging was needed after DBT. Thus 34 of 996 (3%) patients had a change in management triggered by DBT requiring additional imaging. One of these patients was recommended to have short term followup imaging which had not been recommended after FFDM. The remaining 33 patients had 36 additional examinations performed after the addition of DBT. Of the 33 patients, 6 required additional mammogram images, 18 required ultrasound, and 6 patients required both additional mammogram images and ultrasound (12 exams). Biopsies were recommended in 5 patients after DBT alone or based on additional imaging prompted by DBT.

### 3.2. Factors Associated with Management Change

The 996 patients were categorized based on indication for examination as asymptomatic screening symptomatic patients presenting for diagnostic evaluation, or patients recalled from prior screening. Patients who were recalled from screening were significantly more likely to have a management change with the addition of DBT; 24 of the 97 patients (25%) who had DBT as part of recall had a change in management, compared to 14 of the 167 symptomatic patients (8%) and 71 of the 732 asymptomatic patients (10%) (*P* < 0.001). There was no difference in impact of DBT in the screening and symptomatic diagnostic groups; 71 of the 732 patients (10%) who were asymptomatic had a change in management compared to 14 of the 167 symptomatic patients (8%) who had a change in management (*P* = 0.12, 0.19, resp.).

Breast density was evaluated at the time of FFDM. Patients with BI-RADS types 3 and 4 breast density had a significantly higher likelihood of having DBT change management: 68 of 525 (13%) with heterogeneously or extremely dense breasts had a change recommended compared to 41 of 471 (9%) of those with fatty or scattered fibroglandular breast tissue (*P* = 0.03).

Patient age was not associated with an increased likelihood of change in management. For women younger than 50 years old, 52 of 412 (13%) had a change in management recommended, compared to 57 of 584 (10%) for those 50 and older (*P* = 0.15). For women under 50 the most common change in management was reduction in need for additional imaging in 39 of 52 cases (75%).

Although the subgroups are too small to allow for statistically significant data, there was no trend identified associated with identifiable risk factors for breast cancer, such as personal history, family history, prior diagnosis of high risk lesion including atypia or LCIS, or *BRCA* mutation, that were associated with a change in management based on DBT.

### 3.3. Cancer Detection

Of the 996 patients, 19 (2%) were diagnosed with breast cancer (See Table 4). 15 of 18 cancers (83%) were seen on both FFDM and DBT, while 3 of the 18 (17%) were diagnosed on DBT findings alone or after additional imaging generated by DBT (0.3%, 95%CI: 0.1–0.9%) (see Table 3). One cancer was a cutaneous metastatic deposit that was seen neither on FFDM or DBT. Of the 3 patients with cancer detected by DBT, 2 had biopsies based on DBT results alone, while 1 patient had additional imaging triggered by DBT findings and US confirmed a suspicious lesion which was biopsied under US guidance.

Ninety-five biopsies were recommended in the 887 patients without a management change. 81 biopsies were performed with cancer detected in 16 of 81 (20%). Of the 14 patients for whom biopsy was recommended but not performed, 8 were lost to followup, and 6 biopsies were cancelled after repeat imaging performed immediately preceding the biopsy led to cancellation of the recommendation. Of the 6 patients with biopsies cancelled at the time of the procedure, 2 went on to have biopsies after 6 month followup imaging was performed. Both resulted in benign findings.

The addition of DBT led to biopsies in 5 patients with 3 (60%) demonstrating cancer. At surgery, tumor sizes were 1.4 cm, 1.6 cm, and microinvasive (see Table 3).

With FFDM alone, the overall cancer detection rate was 15 of 996 (1.5%). With the addition of DBT the cancer detection rate rose to 18 of 996 (1.8%), an increase of 20% (0.3%, 95%CI: 0.1–0.9%).

### 3.4. Factors Associated with Cancer Detection

Of the 15 patients whose cancers were detected by FFDM and DBT, 6 were seen in the 732 asymptomatic screened patients and thus the cancer detection rate was 1% or 9.6/1000 in this screened population. This high rate of screen detected cancers is expected in this high risk population. Among the 167 symptomatic diagnostic patients, 7 cancers were detected; cancer detection rate was 7 of 167 (4%) or 41.9/1000. Among those recalled from FFDM screening for diagnostic mammography, 2 of 97 (2%) had cancer diagnosed. Of the 3 additional cancers detected by DBT, 1 was in an asymptomatic woman and 2 were in women who were recalled from screening FFDM. Thus the increase in cancer detection rate based on the addition of DBT was 17% for asymptomatic patients, 0% for symptomatic patients, and 100% for those who were recalled from screening.

Of the 15 cancers diagnosed by both FFDM and DBT, cancer detection rate was similar between those with dense breasts (7 of 525, 1.3%) and those with fatty or scattered fibroglandular breasts (8 of 471, 1.7%). The 3 additional cancers diagnosed by DBT alone were all detected in patients with dense breasts.

The 3 patients diagnosed with breast cancer by DBT did not differ significantly from the other 15 diagnosed when comparing patient age, or any other risk factor related to cancer development; however, both were very small groups.

## 4. Discussion

Screening mammography with DBT compared with FFDM alone decreases recall rates while improving cancer detection [14, 20, 21]. Others have investigated DBT in recalled patients and have shown increased reader accuracy [14, 22, 23]. The relative benefit of DBT in patients with dense breasts is of interest, as breast density is a risk factor for breast cancer [24, 25] and reduces mammographic sensitivity [26–28]. The added benefit of DBT in subpopulations of patients, such as those at increased risk for breast cancer or younger women explored in this study, has not been previously described. Furthermore, a large proportion of studies evaluating DBT involve only small numbers of patients, many with fewer than 250 subjects [13].

In a recent important study, constituting the largest DBT screening study to date of women between the ages of 50 and 69, Skaane et al. found a 15% decrease in recall rate using DBT in 12,631 women, and a 27% improvement in the cancer detection rate (from 6.1/1000 to 8/1000) [20]. While results of this study of biennial screening showed that DBT contributed significantly to increased mammographic accuracy, this trial may underestimate the added benefit of DBT in the USA where many women begin annual screening at age 40. In our study, 10% of asymptomatic screening patients had a change in management based on DBT, and the cancer detection rate increased 14%. We did not, however, find a significantly higher cancer detection rate for younger women (<50) based on DBT findings. In the context of diagnostic patients, DBT may replace spot compression views  [14] but does not replace ultrasound. Although not included in this cohort of patients, we have seen cancers that are occult on DBT but visible on ultrasound. This is a known limitation of DBT; DBT is still mammography and shares some of the limitations of FFDM. Cancers can be invisible for many reasons on both DBT and FFDM. Even on DBT some cancers will merge imperceptibly with the surrounding parenchyma and if there is not adjacent fat the cancer can be difficult if not impossible to detect. Other cancers that do not have calcifications or that do not distort the normal breast architecture can be missed on FFDM as well as DBT [29].

While improved cancer detection is an important endpoint in assessing the added benefit of DBT, the reduction in false positives and recall rate is also significant. In 2009, the United States Preventive Services Task Force (USPSTF) reversed their long standing recommendation for yearly mammography in women aged 40–49 largely due to the harms related to false positives and “unnecessary” biopsies [3]. Our findings demonstrated a 9.5% reduction in need for additional imaging among all screened and diagnostic patients and a 25% reduction in recalled patients. 13% of women under age 50 had a change in management, and 75% of these changes resulted in fewer imaging studies performed. It is possible that DBT could lower false positives sufficiently to allow younger women to benefit from mammography with a reduction of the added harms that the USPSTF cautioned against.

The improved accuracy of DBT in those recalled from screening has been demonstrated by multiple authors [30–33] and is confirmed in our study. Patients who were recalled from screening had the most significant increase in cancer detection rate compared to those who were classified as asymptomatic or symptomatic. Our results are similar to those of Skaane et al. [14] who reported that, of 84 women recalled from screening who were called “normal” after a standard mammogram workup, the addition of DBT found 2 additional cancers. This number is similar to ours where 2 of 97 women with cancer would have had a false negative result if only a standard mammogram workup was performed.

In patients at elevated risk for breast cancer due to family history, personal history of breast cancer, or prior biopsy (e.g., for atypia, LCIS, or multiple biopsies), we found no detectible incremental benefit for tomosynthesis compared to FFDM. In contrast, however, our data did indicate that DBT was most beneficial in patients with increased breast density, as the 3 additional cancers detected by DBT were found in patients with heterogeneously or extremely dense breasts. This data conflicts with that of Waldherr et al. who found the breast cancer detection rate to be about equally improved by DBT in those with fatty and dense breasts [30]. Recent legislation in some states has required patient notification of breast density and suggests that patients discuss possible supplemental screening [34, 35] with their healthcare providers. While we are not suggesting that DBT replaces ultrasound or MRI as supplemental screening, given our results DBT may benefit women with dense breasts.

The relative and added benefit of DBT in various subpopulations such as those at increased familial risk for breast cancer has not been previously described. The population studied here represents an example of such a cohort; 32% of patients in our cohort had at least 1 first degree relative with breast cancer, significantly higher than the 7.3% prevalence in the general population [36]. While supplemental MRI screening is established for screening of the highest risk cohorts, optimal screening regimens remain to be established for cohorts at intermediate risk. Our results show a change in management in 10% of asymptomatic women. And while there are no specific risk factors identified that predicted higher likelihood of DBT affecting cancer detection rate or lowering false positive findings in this group at elevated risk, the subgroups analyzed may be too small to demonstrate a benefit. As DBT is more widely evaluated, analysis of its benefits in specific groups of patients based on risk factors, such as those who are *BRCA*-positive, those with atypia or LCIS, and those who have undergone multiple previous biopsies, will be possible.

Overall, we found that DBT increased cancer detection rate by 19% and decreased the need for recall in 8–25%, depending on the indication for DBT.

While our study was prospective and one of the larger series to date, it is nevertheless limited by sample size, and the heterogeneous and self selected nature of the population studied which may make the findings of this study difficult to generalize to the screening or callback settings. Our study population is that of a comprehensive breast center, not a screening center nor a retrospective, enriched reader study. The results therefore, while adding to the current literature, may be difficult to extrapolate. Ultrasound and additional mammographic views were readily available and the radiologists evaluating the FFDM knew that they would be seeing the DBT images. These factors may have impacted management recommendations and readings. However, the ready availability of multimodality imaging led to potentially interesting findings. For example, all the cancers diagnosed by DBT were detected by ultrasound to direct biopsy. This suggests that the incremental benefit of DBT compared to ultrasound screening may be small. Future studies to determine the incremental benefit of DBT compared to FFDM combined with screening ultrasound are needed to resolve this question.

In summary, in this study of the effect of DBT on management in a heterogeneous population, call back rates were reduced while cancer detection was increased, particularly in those with dense breasts. These results are consistent with similar studies in screening and recalled populations but are among the first data available highlighting DBT's impact on clinical management of patients at elevated risk for breast cancer seen in the setting of a comprehensive breast center. Future studies will add to the growing body of knowledge regarding the role of this technology in breast cancer screening and diagnosis and its optimal application.

## Figures and Tables

**Table:** (a)

Number of patients	*n* = 996
Age	
(i) Mean	53.1
(ii) Range	25–87
Breast density	
(i) Fatty	84/996 (8.4%)
(ii) Scattered	387/996 (38.9%)
(iii) Heterogeneously dense	454/996 (45.6%)
(iiii) Extremely dense	71/996 (7.1%)
Risk factors	
Personal history of breast cancer	207/996 (20.8%)
(i) Unilateral breast cancer	198/207 (95.7%)
(ii) Bilateral breast cancer	9/207 (4.3%)
(iii) Breast conserving therapy	142/207 (68.6%)
(iiii)Mastectomy*	68/207 (32.9%)
*3/6 bilateral breast	
cancer patients had both	
BCT and mastectomy	
BRCA positivity	11/996 (1.1%)
ADH	11/996 (1.1%)
LCIS	9/996 (0.9%)
Nonspecified benign biopsy	368/996 (36.9%)
Family history of breast cancer	324/996 (32.5%)
(i) First degree	183/324 (56.5%)
(ii) Second degree	191/324 (59.0%)
(iii) Both	49/324 (15.1%)
(iiii) Not reported	4/996 (1.2%)

*3/9 bilateral breast cancer patients had a unilateral mastectomy and contralateral breast conserving therapy.

**Table:** (b)

Number of patients	*n* = 996
Screening exam	*n* = 711 (71.4%)
(i) Unremarkable breast history	330/711 (46.4%)
(ii) Personal h/o breast cancer	188/711 (26.4%)
(iii) High risk	185/711 (26.1%)
(iiii) Distant h/o breast intervention	8/711 (1.1%)
Diagnostic exam	*n* = 285 (28.6%)
(i) Symptoms	167/285 (58.6%)
(ii) Recalled for additional imaging	97/285 (34.0%)
(iii) Short term followup	21/285 (7.4%)

**Table 2:** Factors affecting change in management.

Factors	Change in management *n* = 109	No change in management *n* = 887	*P* value
Breast density			
(i) Dense	68/109 (62.4%)	457/887 (51.5%)	*P* = 0.034
(ii) Not dense	41/109 (37.6%)	430/887 (48.5%)	
Exam indications			
(i) Screening	72/109 (66.1%)	639/887 (72.0%)	*P* < 0.001
(ii) Diagnostic	37/109 (33.9%)	248/887 (28.0%)	
Prior diagnosis of Atypia or LCIS			
(i) Yes	2/109 (1.8%)	18/887 (2.0%)	*P* = 0.984
(ii) No	107/109 (98.2%)	869/887 (98.0%)	
BRCA mutation			
(i) Yes	1/109 (0.9%)	12/887 (1.4%)	*P* = 0.705
(ii) No	108/109 (99.1%)	875/887 (98.4%)	
Family history of breast cancer			
(i) Yes	40/109 (36.7%)	285/887 (32.1%)	*P* = 0.325
(ii) No	69/109 (63.3%)	602/887 (67.9%)	
Personal history of breast cancer			
(i) Yes	18/109 (16.5%)	189/887 (21.3%)	*P* = 0.244
(ii) No	91/109 (83.5%)	698/887 (78.7%)	
Age			
(i) <50	52/109 (47.7%)	360/887 (40.6%)	*P* = 0.154
(ii) ≥50	57/109 (52.3%)	527/887 (59.4%)	

**Table 3:** Cancers diagnosed.

Age	Exam indication	Breast density: BI-RADS	Cancer detected by	Type of biopsy	Tumor type	Type of surgery	Largest tumor size on excision (mm)
69	Screening	3	FFDM	Stereo	DCIS	Mast	DCIS
75	Diagnostic	2	FFDM	US	IDC	Unk*	Unk*
55	Screening	3	FFDM	US	DCIS	Unk*	Unk*
46	Diagnostic	2	FFDM	US	IDC	BCT	20
49	Diagnostic	2	FFDM	US	ILC	BCT	11
47	Diagnostic	2	FFDM	US	IDC	BCT	5
57	Screening	3	FFDM	Punch bx	IDC	Mast	3
56	Screening	3	FFDM	US	ILC/IDC	Mast	18
50	Diagnostic	2	FFDM	US	IDC	BCT	14
56	Diagnostic	3	FFDM	US	DCIS	BCT	7
47	Screening	2	FFDM	Stereo	DCIS	Mast	DCIS
46	Screening	4	FFDM	US	IDC	Mast	11
69	Screening	2	FFDM	US	IDC	BCT	4
53	Diagnostic	3	FFDM	MRI	IDC	Mast	Unk*
49	Diagnostic	1	FFDM	US	IDC	Neoadj	—
42	Diagnostic	3	FFDM	US	IDC	Mast	18
61	Screening	4	DBT	US	IDC/ILC	BCT	14
66	Diagnostic	3	DBT	US	IDC	BCT	DCIS
59	Diagnostic	3	DBT	US	IDC/lobular features	BCT	16

*Surgery performed at outside institution, unknown surgical pathology.

LEGEND:

Asymp: asymptomatic

Symp: symptomatic

BI-RADS breast density categories:

BI-RADS 1 fatty breasts

BI-RADS 2 scattered fibroglandular elements

BI-RADS 3 heterogeneously dense

BI-RADS 4 extremely dense

IDC: invasive ductal cancer

ILC: invasive lobular cancer

BCT: breast conservation therapy

Unk: unknown.

**Table:** (a)

	Patient 19	Patient 16	Patient 1
Age	61	59	66
History of breast cancer	No	No	No
High risk	Yes	No	No
Risk factor	2° family history	—	—
Symptoms	None	None (call back)	None (call back)
Imaging finding	Spiculated mass	Spiculated mass	Irregular mass
Breast density	Extremely dense	Heterogeneously dense	Heterogeneously dense
Type of biopsy	Ultrasound core	Ultrasound core	Ultrasound core
Focality	Unifocal	Unifocal	Unifocal
Nodal status	1/1 (0.1 cm)	0/1	0/1
Tumor size on excision	14 mm	16 mm	— (residual DCIS only)
Tumor type	IDC/ILC	IDC/lobular features	IDC/DCIS
Grade	Poorly differentiated	Moderately differentiated	Well-moderately differentiated
Molecular subtype (ER/PR/Her2)	+/+/−	+/+/−	+/+/−

**Table:** (b)

Patient	Seen on FFDM	Seen on DBT	Visualized on US	Mass	Imaging size (mm)	Shape	Margin	Calcs	Distribution	Character
1	No	Yes	Yes	Yes	6 mm	Irregular	Irregular	No	—	
2	Yes	Yes	Not done	No	—	—	—	Yes	Group	Linear branching
3	Yes	Yes	Yes	Yes	40 mm	Irregular	Spiculated	No	—	
4	Yes	Yes	Yes	Yes	9 mm	Irregular	Indistinct	No	—	
5	Yes	Yes	Yes	Yes	53 mm	Oval	Irregular	No	—	
6	Yes	Yes	Yes	Architectural distortion	13 mm	Irregular	Irregular	Yes	Group	Punctate
7	Yes	Yes	Yes	Yes*	10 mm	Irregular	Spiculated	No	—	
8****	No	No	No	No	—	—	—		—	
9	Yes	Yes	Yes	Architectural distortion**	15 mm	Irregular	Spiculated	No		
10	Yes	Yes	Yes	Yes	10 mm	Irregular***	Irregular	No		
11	Yes	Yes	Yes	Yes*	11 mm	Round-lobulated	n/a	No		
12	Yes	Yes	Not Done	No	—	—	—	Yes	2 Groups	Pleo- morphic
13	Yes	Yes	Yes	Yes	11 mm	Round-spiculated	n/a	No		
14	Yes	Yes	Yes	Yes	7 mm	Irregular	Spiculated	Yes	Associated	Coarse
15	Yes	Yes	Yes	Yes	3 mm	Oval	Lobulated	No		
16	No	Yes	Yes	Yes	20 mm	Irregular	Spiculated	No		
17	Yes	Yes	Yes	Yes	35 mm	Irregular	Lobulated	No		
18	Yes	Yes	Yes	Yes	4 mm	Irregular	Spiculated	Yes	Segmental	Hetero-geneous
19	No	Yes	Yes	Yes	14 mm	Irregular	Spiculated	No		

*Asymmetry seen on FFDM, mass on DBT only.

**DBT identified a second 8 mm mass with irregular margins.

***Microlobulated mass on DBT.

****Skin lesion was metastatic invasive ductal carcinoma.
